# As Omicron Takes Hold and Other New Variants Arise, COVID-19 Testing Remains the Universally Agreed Tool to Effect Transition From Pandemic to Endemic State

**DOI:** 10.3389/fpubh.2022.883066

**Published:** 2022-04-28

**Authors:** Melissa B. Miller, Eng Eong Ooi, Daniel D. Rhoads, Martin Kulldorff, Danielle E. Anderson, Hyukmin Lee, Sunetra Gupta, Krajden Mel

**Affiliations:** ^1^Department of Pathology and Laboratory Medicine, University of North Carolina School of Medicine, Chapel Hill, NC, United States; ^2^Program in Emerging Infectious Diseases, Duke-NUS Medical School, Singapore, Singapore; ^3^Viral Research and Experimental Medicine Centre, SingHealth Duke-NUS Academic Medical Centre, Singapore, Singapore; ^4^Saw Swee Hock School of Public Health, National University of Singapore, Singapore, Singapore; ^5^Department of Microbiology and Immunology, Yong Loo Lin School of Medicine, National University of Singapore, Singapore, Singapore; ^6^Department of Pathology, Cleveland Clinic Lerner College of Medicine, Case Western Reserve University, Cleveland, OH, United States; ^7^Department of Laboratory Medicine, Cleveland Clinic, Cleveland, OH, United States; ^8^Division of Pharmacoepidemiology and Pharmacoeconomics, Department of Medicine, Brigham and Women's Hospital and Harvard Medical School, Boston, MA, United States; ^9^Department of Microbiology and Immunology, The University of Melbourne at The Peter Doherty Institute for Infection and Immunity, Melbourne, VIC, Australia; ^10^Department of Laboratory Medicine, Severance Hospital, Yonsei University College of Medicine, Seoul, South Korea; ^11^Department of Zoology, University of Oxford, Oxford, United Kingdom; ^12^British Columbia Centre for Disease Control Public Health Laboratory, Vancouver, BC, Canada

**Keywords:** SARS-CoV-2, screening, surveillance, diagnosis, public health, guidelines

## Abstract

The COVID-19 pandemic has caused more than 448 million cases and 6 million deaths worldwide to date. Omicron is now the dominant SARS-CoV-2 variant, making up more than 90% of cases in countries reporting sequencing data. As the pandemic continues into its third year, continued testing is a strategic and necessary tool for transitioning to an endemic state of COVID-19. Here, we address three critical topics pertaining to the transition from pandemic to endemic: defining the endemic state for COVID-19, highlighting the role of SARS-CoV-2 testing as endemicity is approached, and recommending parameters for SARS-CoV-2 testing once endemicity is reached. We argue for an approach that capitalizes on the current public health momentum to increase capacity for PCR-based testing and whole genome sequencing to monitor emerging infectious diseases. Strategic development and utilization of testing, including viral panels in addition to vaccination, can keep SARS-CoV-2 in a manageable endemic state and build a framework of preparedness for the next pandemic.

## Introduction

Severe Acute Respiratory Syndrome Coronavirus 2 (SARS-CoV-2) emerged at the end of 2019, causing a global pandemic with more than 448 million cases and 6 million deaths worldwide to date (1). Omicron is now the dominant variant of SARS-CoV-2, the virus that causes COVID-19. As of March 2022, omicron makes up more than 90% of cases in most countries reporting sequencing data (2). Approximately 65% of the world's population has been vaccinated against SARS-CoV-2 (1) and many have looked hopefully toward a return to pre-pandemic conditions. However, it is becoming increasingly apparent that SARS-CoV-2 will not be eradicated, but will transition to an endemic state (3). In the midst of this transition, new variants of concern continue to arise and challenges in public health remain central on the world stage. One topic of frequent debate has been the role of testing in the diagnosis, screening, and surveillance of COVID-19 (4). Clear public health recommendations on SARS-CoV-2 testing are needed to supplement guidance that has largely been focused on the treatment of COVID-19 (5).

This perspective is the result of a discussion between the authors, who represent thought leadership from a variety of disciplines as well as differing points of view ranging from limited isolation to complete lock-down on how to manage this pandemic. Importantly, all authors agree that careful management of an eventual transition from COVID-19 pandemic to endemicity requires continued use of SARS-CoV-2 testing. We address three critical topics pertaining to the transition from COVID-19 pandemic to endemic: defining the endemic state for COVID-19, highlighting the role of SARS-CoV-2 testing as endemicity is approached, and recommending parameters for SARS-CoV-2 testing once endemicity is reached.

## Defining The Endemic State For COVID-19

The most important driver of the transition from pandemic to endemic will be immunity derived from vaccination or past infection coupled with proper public health control measures. Reported case rates will not necessarily be useful in determining endemicity, as the role of asymptomatic cases and seasonal fluctuations may be a natural part of endemic COVID-19, or may be indicators of concern.

Therefore, we propose a two-criteria framework for endemic COVID-19. First, low annual hospitalization and death rates must be reached. What defines sufficiently low will vary regionally according to demographics, access to resources, healthcare capacity, migration status, and cultural norms. Ideally, the World Health Organization should set out such criteria for its member states and provide technical guidance. Second, low hospitalizations and deaths must be maintained without the need for infection prevention measures in public areas such as facemasks, business closures, or restrictions on events.

## Role of Testing During The Transition From Pandemic To Endemic

While vaccination efforts are the critical driver on the path to COVID-19 endemicity, they alone are insufficient for several reasons. We see vaccine hesitancy in many regions resulting in waning vaccination rates. In the United States, vaccination rates from July 2021 to February 2022 rank among the slowest of the world's seven wealthiest large democracies (6). Additionally, in certain regions there is continued opposition to public health measures such as mask-wearing. This combination of waning vaccination rates and resistance to public health measures results in suboptimal virus control.

Importantly, case rates are not permanently reduced by vaccination alone. In the United Kingdom, the daily COVID-19 case rate exceeds 42,000 per day despite 85.4% of their population aged 12 and up being fully vaccinated (7). Variants of concern can have differential response to the vaccines, with vaccine effectiveness against symptomatic infection by the omicron variant estimated at ~40% and up to 71% after a booster (8). Given these factors, we believe testing will continue to play a crucial role in managing viral spread as we return to normalcy.

Three primary modes of testing have been important during the pandemic and will continue to be utilized—though to different extents—throughout the transition from pandemic to endemic state: diagnostic, screening, and surveillance testing (9). We define diagnostic testing as testing of patients who present with symptoms of acute respiratory illness, screening as testing of asymptomatic individuals in particular settings such as elder care, and surveillance as population-level testing of samples from symptomatic and asymptomatic people. The specific utility of each testing type will be dependent on regional rates of immunity and access to healthcare resources (see Table 1).

**Table 1:** Variations in SARS-CoV-2 testing in regions with differential vaccination/immunity rates and access to healthcare resources.

		**Sufficient access to healthcare resources**	**Limited access to healthcare resources**
High immunity %	Diagnostic	Focus on increasing overall PCR capacity to accommodate SARS-CoV-2 and other molecular diagnoses Increase utilization of and reimbursement for respiratory panel tests	Focus on improving access to SARS-CoV-2 PCR testing and leveraging existing infrastructure and using RADTs to support already existing PCR testing
	Screening	Shift to PCR screening as the positive predictive value of RADT testing will decrease proportionally to decreasing viral loads Prioritize screening of those in contact with at-risk populations	Limit PCR-based screening investment to at-risk populations RADT or isothermal amplification (e.g., SHERLOCK) will be useful in venues with high capacities or that involve border control
	Surveillance	Governments, research institutions, and laboratories should collaborate to create standardized panel-based surveillance programs that will be useful to detect immunity-escaping variants and beyond SARS-CoV-2, including testing best practices and quality assurance methodology	
Low immunity %	Diagnostic	Focus on increasing overall PCR capacity; SARS-CoV-2 should become a standard part of a respiratory panel including influenza A/B and RSV	Focus on improving access to PCR testing specifically for SARS-CoV-2
	Screening	Incentivize screening as a measure to aid reopening and “returning to life” RADT screening will remain useful but may be insufficient in elderly care settings	RADT screening will remain useful but may be insufficient in elderly care settings
	Surveillance	In addition to the steps recommended for high vaccination settings,	
		Incentivize whole-genome sequencing of all SARS-CoV-2 positive tests to support variant surveillance	Conduct variant monitoring via reflex testing of all positives with a mutation panel

*PCR, polymerase chain reaction; RADT, rapid antigen diagnostic test; RSV, respiratory syncytial virus*.

Demand for diagnostic testing will be sustained as immunity increases, but the type of test recommended will change. Specifically, in populations with high immunity, the value of polymerase chain reaction (PCR) tests will increase as the positive predictive value of rapid antigen diagnostic testing (RADT) decreases proportionally to decreasing viral loads (10). Regions with access to sufficient healthcare resources should seek to implement diagnostic PCR testing for SARS-CoV-2 as part of a respiratory panel including influenza A/B and respiratory syncytial virus (RSV). However, PCR panels may not be feasible in resource-restricted low- and middle-income countries (LMIC), where the focus should be on improving access to SARS-CoV-2 PCR testing via mobile PCR platforms and continuing to utilize RADT (11).

Screening will remain crucial particularly while vaccination rates and immunity improve. In addition to protecting the vulnerable, such as nursing home residents and migrant populations, and monitoring the global distribution of variants, screening will enable the comparison of reported infection rates between vaccinated and unvaccinated persons. The impact of immune escape of SARS-CoV-2 variants and the durability of immunity are two variables that are uncertain.

Surveillance testing is a crucially important yet underutilized tool in the transition to a manageable endemic state, particularly in LMICs. For effective surveillance, random samples should be collected consistently in different geographical areas and age groups. When paired with surveillance tests, whole-genome sequencing (WGS) can be powerful but is costly. In regions where the cost may be prohibitive, WGS can be reserved for the identification of “new” variants not identified by PCR panels. WGS will also be important as antiviral therapies become more widely available, to ensure that the antiviral targets have not mutated.

## Differential Value of RADT and PCR Test Modalities

Both RADT and PCR testing are needed during the transition to endemic COVID-19, although with differing applicability. Where feasible, diagnostic testing needs to shift toward PCR tests, which have greater sensitivity and specificity and are superior in diagnosing symptomatic and asymptomatic patients. PCR tests should be used to confirm a negative RADT in high-risk settings where consequences of a false negative result can be severe.

Screening via RADT will continue to provide useful insights in settings with high population density, regions with low immunity and high incidence, and in monitoring temporal and geographic fluctuations, at a potentially very low cost per test with a quick turnaround time. However, RADT are limited in their ability to provide “proof of negativity,” and many entities (e.g., airlines) are increasingly requiring laboratory proof of negativity via PCR testing instead of RADT. One exception to this may be the BinaxNOW RADT, which has specificity close to 100% (12).

The expense of PCR testing compared to RADT is of particular consideration in low-resource settings and highly infectious contexts, as diagnostic labs with limited resources must balance competing needs to fulfill both SARS-CoV-2 and other testing needs. Therefore, RADTs or isothermal amplification tests (e.g., specific high-sensitivity enzymatic reporter unlocking [SHERLOCK]) can be used to support PCR, especially in situations where the testing demand exceeds the supply of PCR tests or when turnaround time is critical. However, RADT shortages, such as those being seen in the US, may also necessitate greater reliance on lab-based PCR tests (13).

## Testing During Endemicity

Incentives for testing should continue once COVID-19 reaches an endemic equilibrium to raise awareness and education regarding the role of testing in protecting vulnerable populations. To avoid financial burden on individuals, tests need to be heavily or fully subsidized by governments, employers, or medical insurance. For example, if workers are required by their employer to be tested regularly, they should not have to pay for the tests themselves. The World Health Organization has established the Access to COVID-19 Tools Accelerator (ACT-A) to provide tests to LMIC; however, funding for ACT-A is an ongoing challenge (14). Multinational programs such as ACT-A could also play a role in standardizing testing protocols, streamlining data reporting, and disseminating information on best practices (15).

International travel will continue to be a useful opportunity for testing during endemic COVID-19. Requiring tests for travelers has been demonstrated to be a successful way to monitor variants such as omicron that may pose a challenge to herd immunity (16). Testing of travelers from regions where there is increased disease prevalence or variants of interest/concern can potentially reduce transmission (17). In addition, travel testing can monitor global disease prevalence and assess geographic and longitudinal trends (18).

Discrimination between SARS-CoV-2 and other respiratory viruses will continue to be important in optimizing patient care during endemic COVID-19. Therefore, multiplex PCR assays to detect SARS-CoV-2, influenza A/B, and RSV should become a routine part of clinical management of patients who present with acute respiratory illness (19). Surveillance testing for antibodies could also be useful to monitor if population immunity is waning over time. Studies have shown that the titer of anti-SARS-CoV-2 IgG antibody is detectable up to 15 months after recovery from COVID-19 (20).

## Quality Assurance and Looking Ahead To Future Pandemics

Quality assurance programs for SARS-CoV-2 tests are necessary due to global variability in test performance. Approval of a test for use during the pandemic has not necessarily equated to a high-quality test. For example, as of July 15, 2021, the US Food and Drug Administration had determined that 289 SARS-CoV-2 test kits should no longer be used or distributed due to failure to meet regulatory requirements (13). However, government intervention should be carefully designed so as not to limit the potential positive impacts of innovation (21).

In preparation for future pandemics and other infectious disease outbreaks, governments and global leaders need to invest in testing capacity and quality and also support the ability to rapidly scale testing when needed. We should capitalize on the current momentum to increase capacity for PCR-based testing and WGS to monitor emerging infectious diseases. SARS-CoV-2 is not going to exit the world stage soon, but strategic development and utilization of testing, including viral panels in addition to vaccination, can keep it in a manageable endemic state and begin preparing us for the next pandemic.

## Conclusions

In this perspective, we have provided new insights into the evolving public health dialogue around COVID-19 by putting forth a definition of endemic COVID-19, outlining the role of SARS-CoV-2 testing throughout the transition from pandemic to endemic, and recommending parameters for testing during endemicity. Both rapid antigen and PCR tests will remain important testing modalities, with particular need for viral panels that include SARS-CoV-2 during endemicity.

## Data Availability Statement

The original contributions presented in the study are included in the article, further inquiries can be directed to the corresponding author.

## Author Contributions

To prepare this article, two global advisory boards were sponsored by Thermo Fisher Scientific under their Thought Leader Advisory Program and moderated by Boston Strategic Partners. The authors represent thought leadership from a variety of disciplines and share differing points of view on how to manage this pandemic. This manuscript summarizes their collective views on how testing may be conducted as we approach a state of endemicity. All authors contributed to the manuscript. None of the authors received financial compensation for writing this article.

## Conflict of Interest

The authors declare that the research was conducted in the absence of any commercial or financial relationships that could be construed as a potential conflict of interest.

## Publisher's Note

All claims expressed in this article are solely those of the authors and do not necessarily represent those of their affiliated organizations, or those of the publisher, the editors and the reviewers. Any product that may be evaluated in this article, or claim that may be made by its manufacturer, is not guaranteed or endorsed by the publisher.
